# A Novel *Lentinula edodes* Laccase and Its Comparative Enzymology Suggest Guaiacol-Based Laccase Engineering for Bioremediation

**DOI:** 10.1371/journal.pone.0066426

**Published:** 2013-06-14

**Authors:** Kin-Sing Wong, Man-Kit Cheung, Chun-Hang Au, Hoi-Shan Kwan

**Affiliations:** School of Life Sciences, The Chinese University of Hong Kong, Shatin, Hong Kong; Instituto de Tecnologica Química e Biológica, UNL, Portugal

## Abstract

Laccases are versatile biocatalysts for the bioremediation of various xenobiotics, including dyes and polyaromatic hydrocarbons. However, current sources of new enzymes, simple heterologous expression hosts and enzymatic information (such as the appropriateness of common screening substrates on laccase engineering) remain scarce to support efficient engineering of laccase for better “green” applications. To address the issue, this study began with cloning the laccase family of *Lentinula edodes*. Three laccases *perfectio sensu stricto* (Lcc4A, Lcc5, and Lcc7) were then expressed from *Pichia pastoris*, characterized and compared with the previously reported Lcc1A and Lcc1B in terms of kinetics, stability, and degradation of dyes and polyaromatic hydrocarbons. Lcc7 represented a novel laccase, and it exhibited both the highest catalytic efficiency (assayed with 2,2′-azino-bis(3-ethylbenzothiazoline-6-sulfonic acid) [ABTS]) and thermostability. However, its performance on “green” applications surprisingly did not match the activity on the common screening substrates, namely, ABTS and 2,6-dimethoxyphenol. On the other hand, correlation analyses revealed that guaiacol is much better associated with the decolorization of multiple structurally different dyes than are the two common screening substrates. Comparison of the oxidation chemistry of guaiacol and phenolic dyes, such as azo dyes, further showed that they both involve generation of phenoxyl radicals in laccase-catalyzed oxidation. In summary, this study concluded a robust expression platform of *L. edodes* laccases, novel laccases, and an indicative screening substrate, guaiacol, which are all essential fundamentals for appropriately driving the engineering of laccases towards more efficient “green” applications.

## Introduction

Laccases (benzenediol:oxygen oxidoreductase, E.C.1.10.3.2) are multi-Cu oxidases which demonstrate a high relevance to various environment-friendly applications, such as bioremediations and biorefinery [1], [2], [3]. The enzymes catalyze a single-electron oxidation of small aromatic substrates with a concomitant reduction of molecular oxygen into water [1], [4], [5]. This simple requirement and versatile catalysis have resulted in an exponential growth of interest both in academia and industry [1], [6]. Novel and engineered laccases have thus been emerging to find an outstanding candidate with superior performance on “green” applications [6], [7], [8].

White-rot basidiomycetes are a resourceful sink of high-redox-potential laccases (>0.7 V) that are good starting candidates of directed evolution [6], [9], [10]. *Lentinula edodes*, a popular edible mushroom in Asia, is a selective lignin-degrading white-rot basidiomycete recruiting laccases and Mn peroxidases, but no lignin peroxidase, to break down lignin [11]. With reference to the genome sequence of *L. edodes*, we had recently established a simple yeast expression platform (*Pichia pastoris*) to produce two allelic forms of recombinant *L. edodes* laccase, namely, Lcc1A and Lcc1B [2]. This platform, together with the genomic information, has enabled a comprehensive investigation into the entire laccase family in *L. edodes* regardless of the complicated purification of individual isozymes from the native host. Novel enzymes could be identified unambiguously, and comparative analyses between expressed candidates will further advance our knowledge on laccase enzymology and support protein engineering.

Laccases demonstrate loose substrate specificity, and they can catalyze the oxidation of a number of benchmark substrates such as 2,2′-azino-bis(3-ethylbenzothiazoline-6-sulfonic acid) (ABTS), catechol (CAT), L-3,4-dihydroxyphenylalanine (DOPA), 2,6-dimethoxyphenol (DMP), guaiacol (GUA) and syringaldazine (SGZ) (Fig. S1). Among them, ABTS is a non-phenolic substrate which is most commonly recruited for assaying laccase activity due to its intrinsic advantages of pH independence and the high molar extinction coefficient of its oxidized product (ABTS^+•^, ε_420nm_ = 36,000 M^−1^ cm^−1^) [12], [13]. Oxidation of ABTS, in the absence of H_2_O_2_, thus has become a common screening criterion in the molecular evolution of laccases [6], [7], [8]. Either DMP or SGZ, two phenolic substrates, were frequently employed to supplement the screening even though similar trends with ABTS were usually observed [6], [10], [14]. Nevertheless, the goal of laccase engineering is not to achieve a candidate with high catalytic activity/stability on benchmark substrates but more importantly to achieve one on realistic application substrates, such as synthetic dyes and polyaromatic hydrocarbons (PAHs). However, the demonstration of the correlation between oxidation of these popular benchmark substrates and the catalysis on application substrates still remains to be established [13]. Furthermore, these pollutants are usually present as a complex mixture in contaminated effluent and soil [15], [16], [17]. Thus, a detailed comparison of the indication ability among benchmark substrates on multiple application substrates is necessary to justify the substrate choice to drive the engineering of laccase towards better “green” applications.

This study aimed to provide new laccases and useful resources to support laccase engineering. We report herein the cloning of the laccase family of *L. edodes* and the successful expression of three of them from *P. pastoris*, including one novel enzyme (Lcc7) and two homologs of Lcc4 and Lcc5, as reported by a Japanese group [18], [19]. Together with the previously reported Lcc1A and Lcc1B, the comparative enzymology of a spectrum of five recombinant *L. edodes* laccases highlighted hotspots in primary amino acid sequences, and it also enabled a comprehensive analysis of the correlation between laccase activities on benchmark substrates and on application substrates. An indicative benchmark substrate, GUA, was suggested, and it would represent a relevant “direction” for the molecular evolution of laccases for realistic “green” applications.

## Materials and Methods

### Gene Prediction and Molecular Cloning of *L. edodes* Laccase Family

Protein-coding genes were predicted from the genome sequence of a monokaryon *L. edodes* L54-A. Laccase candidate genes were identified from the alignments with the entries in the FOLy database [20], followed by manual curation in accordance with the sequence features described by Kumar *et al*. [21] and Hoegger *et al*. [22]. The genes were then amplified from a cDNA library derived from the reverse transcription of mycelial RNA of a dikaryon *L. edodes* L54, as described previously [2]. Specific primers for the molecular cloning of individual genes are tabulated in Table S1. Verified genes were restricted by appropriate endonucleases (New England Biolabs), ligated into pPIC3.5K (Invitrogen), electroporated into *P. pastoris* GS115 (Mut^+^, His^−^), and verified in accordance with Wong *et al*. [2].

### Heterologous Expression and Purification of Lcc4A, Lcc5, and Lcc7

The production of Lcc4A, Lcc5, and Lcc7 followed the previously described method [2], with minor modifications. The best integrants were subject to fed-batch fermentations in a 5-L continuous stirred tank reactor. The harvested cell-free media were first concentrated and diafiltrated with 10 mM sodium phosphate (pH 7) by using a cassette-style tangential flow ultrafiltration system equipped with an Ultracel Membrane (molecular weight cut-off = 30,000, Millipore). A concentrate harboring Lcc4A or Lcc7 was subject to (NH_4_)_2_SO_4_ precipitation at 30% saturation, while a concentrate with Lcc5 was precipitated at 40% saturation of (NH_4_)_2_SO_4_. After pH adjustment to 7, precipitate-free samples were applied to a Phenyl Sepharose column (40 mL, GE Healthcare) equilibrated with 10 mM sodium phosphate (pH 7) either with 30% or 40% saturation of (NH_4_)_2_SO_4_. The column was then washed before elution of bound protein by a decreasing gradient of (NH_4_)_2_SO_4_. Active fractions were then pooled, concentrated by ultrafiltration using a stirred cell equipped with a YM-10 membrane disc (molecular weight cut-off = 10,000, Millipore), diafiltrated with 10 mM sodium phosphate (pH 7), and stored at 4°C for subsequent analyses. The laccase activity was measured by following the formation of ABTS^+•^ spectrophotometrically at 420 nm (ε_420_ = 36,000 M^−1^cm^−1^) in a standard assay containing 1 mM ABTS (Sigma) in 1×McIlvaine buffer (pH 4) at 30°C. One unit (U) of enzyme activity was defined as the number of µmol of ABTS^+•^ formed in one minute under the above reaction conditions. The zymogram was stained with 1 mM ABTS [23], while the enzyme purity was evaluated by sodium dodecyl sulfate polyacrylamide gel electrophoresis (SDS-PAGE). Protein concentration was determined by the bicinchoninic acid method [24] using bovine serum albumin as the standard.

### Characterizations of Lcc4A, Lcc5, and Lcc7

The apparent *K_m_* (*K_m,app_*) and apparent *V_max_* (*V_max,app_*) values of Lcc4A, Lcc5, and Lcc7 on ABTS, CAT, DOPA, DMP, GUA, SGZ, and tyrosine (TYR) were determined at standard condition containing 1×McIlvaine buffer (pH 4) at 30°C. Stability analyses against temperature, pH, and organic solvents were carried out in accordance with previously reported methods under the standard assay condition using ABTS as the substrate [2].

The decolorization of eight synthetic dyes [methyl red (MR), reactive orange 16 (RO16), Coomassie brilliant blue R-250 (CBBR), bromophenol blue (BPB), crystal violet (CV), indigo carmine (IC), Naphthalo blue black (NBB), and Remazol brilliant blue R (RBBR)], and the degradation of three PAHs [naphthalene (NAP), anthracene (ANT), and benzo[*a*]anthracene (BaA)] by Lcc4A, Lcc5, and Lcc7 were examined both in the absence and the presence of mediators [1-hydroxybenzotriazole (HBT) or 2,2,6,6-tetramethylpiperidine-1-oxyl (TEMPO)]. The amount of enzyme and procedures were essentially the same as the previous report of Lcc1A and Lcc1B in order to allow fair comparisons [2].

### Bioinformatic and Phylogenetic Analyses of Lcc4A, Lcc5, and Lcc7

Amino acid sequences of Lcc4A, Lcc5, and Lcc7 were *in silico* translated from the cloned sequences by Transeq (http://www.ebi.ac.uk/Tools/emboss/transeq/index.html). Potential *N*-glycosylation sites (Asn-X-Ser/Thr) were proposed in accordance with Rodgers *et al*. [1]. SignalP 4.0 (http://www.cbs.dtu.dk/services/SignalP/) was employed to identify the signal peptide cleavage sites. Amino acid sequences were aligned by ClustalW2 (http://www.ebi.ac.uk/Tools/msa/clustalw2/). The hydrophobicity of substrate binding loops was computed by HydroMCalc (http://www.bbcm.univ.trieste.it/~tossi/HydroCalc/HydroMCalc.html). Homology models were constructed with respect to relevant crystal structures by using an automated protein homology modeling server “SWISS-MODEL” (http://swissmodel.expasy.org/).

In addition, the amino acid sequences of laccases from the genome of 11 white-rot basidiomycetes were retrieved from the JGI website (http://genome.jgi.doe.gov/programs/fungi). Only the sequences which perfectly matched the four laccase signatures (*i.e*., laccase *perfectio sensu stricto*) were selected for phylogenetic analyses. Redundant sequences with 100% identity were also removed. Then, remaining sequences were aligned with those from our *L. edodes* laccases by using the einsi algorithm of MAFFT version 6.864 [25]. Conserved regions of the alignment were extracted by using Gblocks 0.91 [26] with minimum block length set to 5 and an allowance of half gap positions. Maximum likelihood phylogenetic tree was built by using PhyML 3.0 [27] with the LG substitution model, a fixed proportion of invariable sites, estimated Gamma distribution parameter and the best of the NNIs and the SPRs tree topology searching approach. Statistical supports for nodes were obtained from 100 bootstrapping replicates. The tree was rooted by the use of *Chaetomium globosum* and *Neurospora crassa* laccases, which acted as outgroups and were displayed by using MEGA5 [28].

### Correlation Analyses between Activity on Benchmark Substrates, Dye Decolorization, and PAH Degradation

The correlation analyses between the catalysis of Lcc1A, Lcc1B, Lcc4A, Lcc5, and Lcc7 on benchmark substrates (specific *V_max,app_*) and on dye decolorization (initial decolorization rate) and PAH degradation (24-hour degradation %) were examined. The performance of laccase-HBT and laccase-TEMPO systems were also included in the correlation analyses. Catalysis of any pair of benchmark substrate and application substrate which demonstrated a correlation coefficient (C) with a magnitude ≥0.9 was considered to be a high association, whereas that with a magnitude of between 0.5 and 0.9 was considered to be a fair association. The correlation was further displayed by a hierarchical clustered heat map generated by MeV v4.8 (http://www.tm4.org/).

## Results and Discussion

### Molecular Cloning of *L. edodes* Laccases Identified Novel Enzymes

Ten laccase genes were cloned from the transcriptome of *L. edodes*, with two of them presenting allelic forms. Sequence analyses categorized five isozymes as *perfectio sensu stricto*. As a result, 12 laccase gene sequences (10 laccases +2 allelic forms) were deposited into GenBank, and their cloning results are summarized in Table 1. The clones covered all known *L. edodes* laccases archived in GenBank with the exception of Lcc6, which was a direct submission without any characterization (Table 1). No known enzymes from *L. edodes* showed a high homology to Lcc7– Lcc11 and hence they were considered to be novel.

**Table 1:** Molecular cloning of laccase family of *L. edodes* L54.

Isozyme	Primers^a^	GenBankaccession	No. of intron	No. of amino acid residue	References (remarks)
Lcc1^b^	Lcc1-F, Lcc1-R	JN607250, JN607251^d^	16	518	[2^e^, 11, 45^e^, 46^e^]
Lcc2^b^	Lcc2-F, Lcc2-R	JX879726	13	533	[47]
Lcc3	Lcc3-F, Lcc3-R	JX879727	11	547	(Sequence archived without relevant literature.)
Lcc4^b^	Lcc4-F, Lcc4-R	JX879728, JX879729^d^	16	527	[18, 19^e^] (Originally misnamed as Lcc2.)
Lcc5^b^	Lcc5-F, Lcc5-R	JX879730	20	515	(Sequence archived without relevant literature.)
Lcc6	N/A^c^	N/A	N/A	N/A	(Not identified from the genome. Sequence archived without relevant literature.)
Lcc7^b^	Lcc7-F, Lcc7-R	JX879731	12	560	This study
Lcc8	Lcc8-F, Lcc8-R	JX879732	23	528	This study
Lcc9	Lcc9-F, Lcc9-R	JX879733	15	524	This study
Lcc10	Lcc10-F, Lcc10-R	JX879734	9	631	This study
Lcc11	Lcc11-F, Lcc11-R	JX879735	9	596	This study

a Sequences were tabulated in Table S1.

b Laccase *perfectio sensu stricto.*

c Not applicable.

d Allelic forms.

e Heterologous expression.

**Figure 1:**
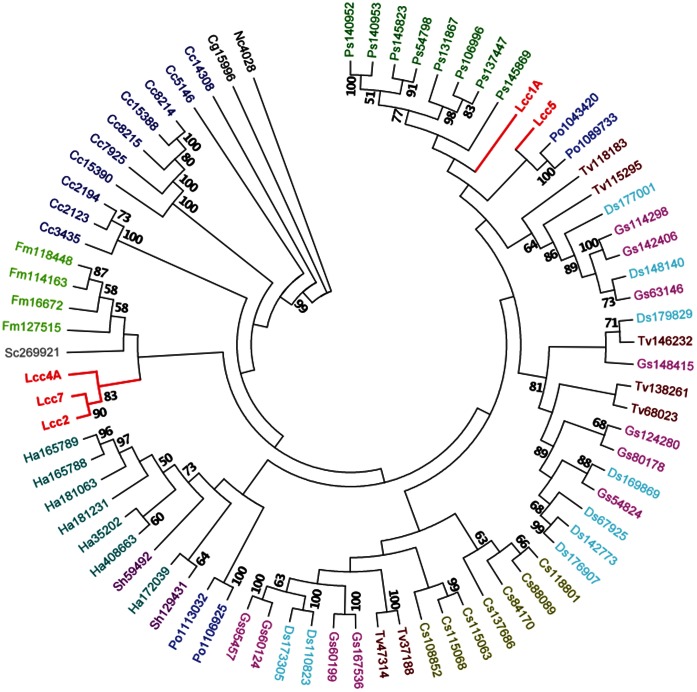
A rooted maximum likelihood phylogenetic tree of basdiomycete laccases *perfectio sensu stricto*. Laccases of *L. edodes* are in red, whereas others are individually colored. Numbers at nodes are bootstrap percentages (≥50%) from 100 replicates. Branch lengths here did not represent evolutionary changes. The tree was rooted by using two ascomycetous sequences [*Chaetomium globosum* (Cg) and *Neurospora crassa* (Nc)]. Cc: *Coprinopsis cinerea*; Fm: *Fomitiporia mediterranea*; Sc: *Schizophyllum commune*; Ha: *Heterobasidion annosum*; Sh: *Stereum hirsutum*; Po: *Pleurotus ostreatus*; Gs: *Ganoderma spp.*; Ds: *Dichomitus squalens*; Tv: *Trametes versicolor*; Cs: *Ceriporiopsis subvermispora*; Ps: *Punctularia strigosozonata*. Numbers following the abbreviations refer to JGI protein IDs in the respective fungal genome.

In fact, the presence of multiple laccase isozymes is not uncommon in white-rot basidiomycetes [1], [4]. The existing categorization of laccases *sensu stricto* requires a combination of sequence and phylogenetic analysis [21], [22]. This study took a more stringent but straightforward approach in that the laccases which perfectly matched the signature sequences were classified as *perfectio sensu stricto*. Phylogenetic analyses of laccase *perfectio sensu stricto* further revealed that *L. edodes* laccases, similar to those of *Pleurotus ostreatus*, separated into distant branches (Fig. 1), thereby suggesting that their occurrence was a result of ancient gene duplication. However, their co-expression in the native host precludes efficient individual analyses and thus a robust heterologous expression platform is a prerequisite to produce discrete laccases and to substantiate protein engineering [29], [30]. Among the 12 laccase genes in *L. edodes*, we expressed Lcc4A, Lcc5, and Lcc7 individually, in addition to the previously reported Lcc1A and Lcc1B, from the heterologous host *P. pastoris* for detailed characterizations. The successful expression justified the high robustness of our yeast expression platform in the production of *L. edodes* laccases. Together with the devised simple purification procedures, this system had made practical the detailed comparative enzymology of *L. edodes* laccases and protein engineering.

### Sequence Alginments and Comparisons of Enzymatic Properties between Recombinant Laccases Suggested “Hotspot” Amino Acid Residues

**Figure 2:**
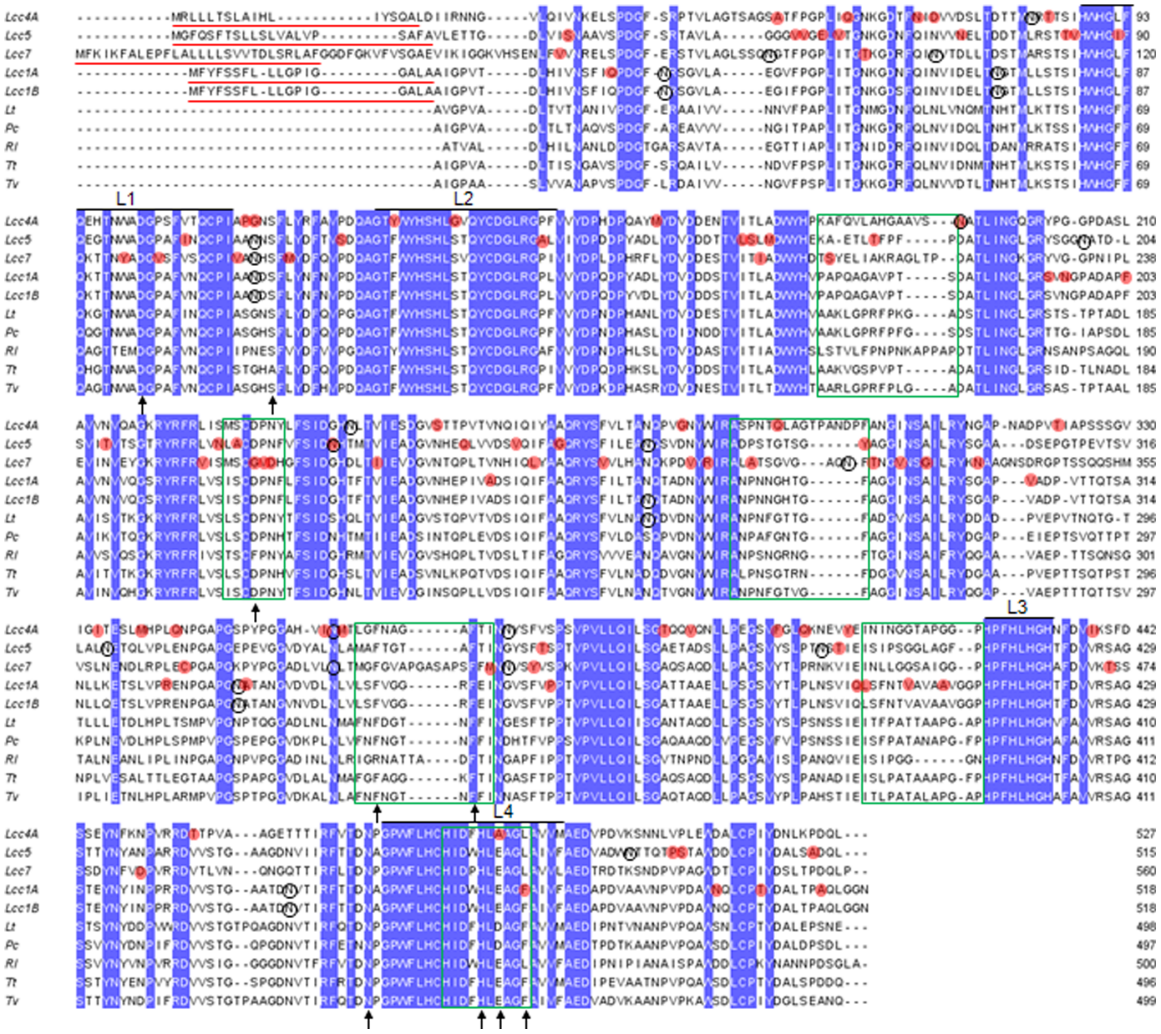
Amino acid sequence alignments of recombinant laccases of *L. edodes* with other fungal laccases. Lt: *Lentinus tigrinus* (PDB: 2QT6); Pc: *Pycnoporus cinnabarinus* (PDB: 2XYB); Rl: *Rigidopours lignosus* (PDB: 1V10); Tt: *Trametes trogii* (PDB: 2HRG); Tv: *Trametes versicolor* (PDB: 1GYC). Predicted signal peptides are red-underlined. Conserved residues are blue-shaded. Unique residues between Lcc1A, Lcc4A, Lcc5, and Lcc7 are red-shaded. Predicted N-glycosylation sites of recombinant laccases are black-circled. Signature sequences (L1–L4) of fungal laccases are annotated. Substrate-binding loops are green-boxed. Residues with known functions (reviewed by Giardina *et al*. [4]) are arrowed.

Amino acid sequence alignments with other fungal laccases are depicted in Figure 2. The signal peptide of the five recombinant enzymes displayed multiple-Leu in spite of length polymorphism. The hydrophobicity of their substrate binding loops was in the order of Lcc5> Lccc1A = Lcc1B>Lcc4A>Lcc7. Furthermore, the sequences revealed five to seven potential *N*-glycosylation sites in the recombinant laccases. Unique substitutions between Lcc1A, Lcc4A, Lcc5, and Lcc7, including those in the signature sequences and in the substrate binding loops, were highlighted (Fig. 2). Indeed, three of them covered residues with known functions: Phe479 of Lcc1A and Lcc1B for hydrophobicity of substrate binding pocket to increase redox potential; Ala488 of Lcc4A for hydrogen bond formation to increase redox potential; Gly259 of Lcc7 for pH dependence of enzyme [4]. Further site-directed mutagenesis studies will help verify their roles and provide insights into the sequence–function relationship of fungal laccases.

Similar to the previously reported Lcc1 [2], Lcc4 also possessed two allelic genes encoding Lcc4A and Lcc4B, but only the former one resulted in an active recombinant laccase. Although sequence alignments revealed a 97% homology between their amino acid sequences, there were four substitutions in Lcc4B lying on conserved residues (G218D, G304A, N306S, and S307G) in respect of its active allelic counterpart (Fig. S2). These substitutions located in the D2 cupredoxin-like domains, but not one was close to the substrate binding pocket nor T1-Cu, as inspected from the homology model (data not shown). However, the latter three substitutions caused a loss of hair pin structure, and this was plausibly detrimental to the activity of Lcc4B. The crucial functional role of such hair pin structure remains opaque and it warrants further investigations.

**Figure 3:**
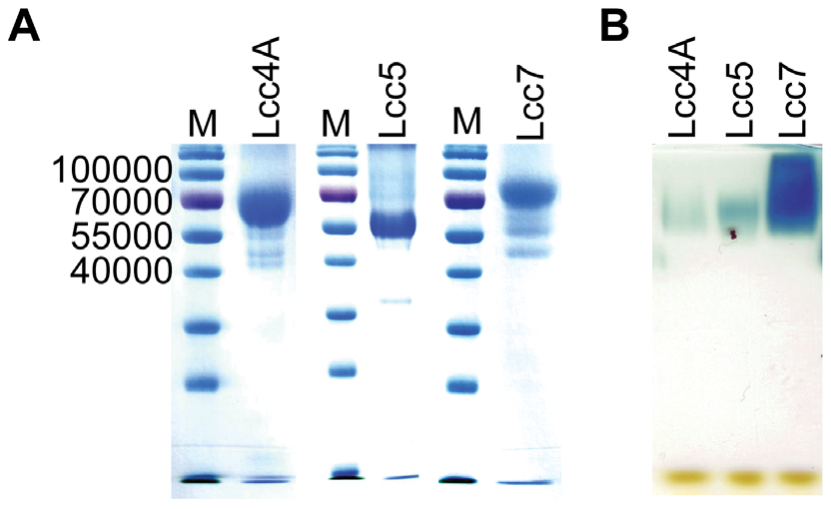
Electrophoretic analyses of Lcc4A, Lcc5, and Lcc7. (A) SDS-PAGE of recombinant laccases. Proteins were denatured before being resolved in 12% (w/v) SDS-PAGE followed by CBBR staining. (B) Zymogram of recombinant laccases. Native proteins were resolved in 12% (w/v) SDS-PAGE followed by an activity staining with 1 mM ABTS in accordance with Srinivasan *et al*. [23].

The expression and purification of Lcc4A, Lcc5, and Lcc7 employed the practical strategy devised from Lcc1A and Lcc1B with minor modifications [2]. It allowed the robust production of discrete isozymes with a comparable quality (Fig. 3). The purified Lcc4A, Lcc5, and Lcc7 demonstrated a specific activity of 0.5 U/mg, 1.5 U/mg, and 11 U/mg, respectively, and their laccase identity was further confirmed by a zymogram (Fig. 3). Their kinetic parameters on various benchmark substrates and stability were assessed (Table 2 and Table 3). All three recombinant laccases were active on SGZ in addition to ABTS, and they did not catalyze the oxidation of TYR, thereby ruling out any ambiguity of tyrosinase contamination [12], [13]. Generally, the enzymes behaved differently in terms of kinetics and stability, and they are individually discussed below.

**Table 2:** Kinetic parameters^a^ of recombinant laccases on benchmark substrates (mean ± SD).

	Lcc4A			Lcc5			Lcc7			Lcc1A			Lcc1B		
Substrate	*K_m_* (µM)	*V_max_* (U mg^−1^)^b^	*V_max_*/*K_m_* (U mg^−1^ M^−1^)^b^	*K_m_* (µM)	*V_max_* (U mg^−1^)^b^	*V_max_*/*K_m_* (U mg^−1 ^M^−1^)^b^	*K_m_* (µM)	*V_max_* (U mg^−1^)^b^	*V_max_*/*K_m_* (U mg^−1 ^M^−1^)^b^	*K_m_* (µM)	*V_max_* (U mg^−1^)^b^	*V_max_*/*K_m_* (U mg^−1 ^M^−1^)^b^	*K_m_* (µM)	*V_max_* (U mg^−1^)^b^	*V_max_*/*K_m_* (U mg^−1 ^M^−1^)^b^
ABTS	11.2±0.9	0.61±0.01	(5.44±0.31)×10^4^	325±47	2.26±0.36	(6.94±0.16) ×10^3^	36.3±1.9	19.7±0.6	(5.43±0.23) ×10^5^	14.3±1.9	2.65±0.65	(1.92±0.75)×10^5^	42.5±10.3	5.61±2.26	(1.28±0.22)×10^5^
CAT	255±76	0.91±0.11	(3.72±0.75)×10^3^	6280±680	2.65±0.10	(4.24±0.31) ×10^2^	38800±5300	0.53±0.05	13.8±0.08	2030±140	0.62±0.13	(3.08±0.85)×10^2^	3540±230	0.94±0.31	(2.69±0.95)×10^2^
DMP	26.0±2.4	0.26±0.03	(1.00±0.03)×10^4^	113±8	0.60±0.03	(5.30±0.28) ×10^3^	12000±1000	1.73±0.09	(1.44±0.05) ×10^2^	120±4	1.08±0.24	(8.94±1.79)×10^3^	115±17	1.41±0.52	(1.20±0.29)×10^4^
DOPA	1040±150	1.07±0.10	(1.03±0.05)×10^3^	10500±700	1.66±0.15	(1.58±0.07) ×10^2^	n.a.^c^	n.a.	n.a.	4550±570	0.49±0.19	(1.06±0.28)×10^2^	5520±850	0.65±0.15	(1.22±0.45)×10^2^
GUA	648±105	0.16±0.01	(2.46±0.27)×10^2^	1270±90	0.32±0.01	(2.57±0.17) ×10^2^	8630±2120	0.18±0.05	21.4±0.3	943±58	0.73±0.13	(7.85±1.72)×10^2^	913±68	0.96±0.25	(1.07±0.35)×10^3^
SGZ	n.d.^d^	0.09±0.01	n.d.	n.d.	0.02±0.00	n.d.	n.d.	2.52±0.81	n.d.	n.d.	0.98±0.20	n.d.	n.d.	0.75±0.07	n.d.
TYR	n.a.	n.a.	n.a.	n.a.	n.a.	n.a.	n.a.	n.a.	n.a.	n.a.	n.a.	n.a.	n.a.	n.a.	n.a.

a Apparent values. Parameters of Lcc1A and Lcc1B were obtained from our previous study [2].

b One U was defined as the number of µmol of respective oxidized product formed in one minute under standard assay condition.

c No measurable activity.

d Not determined.

**Table 3:** Effects of temperature, pH, and co-solvents on recombinant laccases.

					IC50^d^ (v/v %)			
Laccase	T_m_ a (°C)	t_1/2,50C_ b (min.)	Stable pH^c^	pH optimum	ACE	ACN	EtOH	MeOH
Lcc4A	49	28	2–8	2.5	42	30	34	28
Lcc5	33	<15	3–6.5	2.5	20	18	18	22
Lcc7	57	>120	3.5–5	3	25	17	22	24
Lcc1A	47	<15	2–8	2	30	36	30	28
Lcc1B	47	<15	2–8	2	30	36	30	26

a T_m_ was defined as the temperature that 30-minute incubation led to 50% loss of enzyme activity. Assays were performed in 1×McIlvanie buffer (pH 4) by using 1 mM ABTS at 30°C (Fig. S3).

b t_1/2,50C_ was defined as the incubation time at 50°C that caused 50% loss of enzyme activity. Assays were performed in 1×McIlvanie buffer (pH 4) by using 1 mM ABTS at 30°C (Fig. S3).

c Defined by residual activity ≥80%. Assays were performed in 1×McIlvanie buffer (pH 2–8) by using 1 mM ABTS at 30°C (Fig. S6).

d Half maximal inhibitory concentration (IC50) was defined as the concentration of co-solvent that caused 50% loss of enzyme activity. Assays were performed in 1×McIlvanie buffer (pH 4) by using 1 mM ABTS at 30°C in the presence of respective solvent (Fig. S4).

Lcc7 was a novel laccase and it represented the most active (when assayed with ABTS) and thermostable isozyme. Its catalytic efficiency, T_m_ and t_1/2,50C_ were threefold, 10°C and >110 minutes higher/longer than those of Lcc1A, respectively (Table 2, Table 3 and Fig. S3). Compromises were observed from its catalytic efficiency and substrate affinity towards phenolics (CAT, DMP, and GUA), stability at pH <3.5, and to organic solvents (Table 2, Table 3 and Fig. S4). Failure in catalysis on DOPA and very low activity on CAT proposed subtle contribution of native Lcc7 in physiological melanin synthesis in *L. edodes* [18]. Substitution of a conserved Asp residue to Gly259 was observed from the substrate binding moiety of Lcc7 (Fig. 2). This Asp residue, while well conserved in laccases from basidiomycetes, provides a carboxylic function at pH <5 for interaction with arylamines and phenolics [31], [32]. Since the current standard assay was performed at pH 4, the missing carboxylic function of Lcc7 can be a possible reason for its low substrate affinity to phenolic substrates (Table 2). Camarero *et al*. [6] highlighted five amino acid substitutions in a laboratory-evolved *Pycnoporus cinnabarinus* laccase that led to a 14-fold improvement of *k_cat_* towards ABTS. Three of the corresponding residues in Lcc7 (Asp261, Gly389, and Asn405) also differed from the other recombinant laccases (Fig. 2). Taken together, these hotspot substitutions represent interesting candidates for future studies to delineate their structural/functional roles. Furthermore, the elongation (V392-P399) and the least hydrophobicity of the substrate binding loops of Lcc7 were also new cues to subsequent investigations. Indeed, our preliminary homology modeling of Lcc7 suggested that the elongated loop resided at the entrance of the substrate binding pocket (Fig. S5) and its functional role will be examined by constructing a deletion mutant.

Lcc4A exhibited almost an order higher catalytic efficiency toward CAT and DOPA compared to the other four recombinants (Table 2). This was consistent with its native role in melanin synthesis. This is because both CAT and DOPA are building blocks of fungal melanin [18]. The current isolation of the transcript of Lcc4 from mycelial RNA suggests that its transcription began before the development of fruiting body. Both the thermostability and the pH dependence of recombinant Lcc4A were similar to those of native protein, except that a broader range of pH stability was observed (Table 3 and Fig. S6) [18]. Differences of strains and post-translational modifications could contribute to the discrepancy. Indeed, Yano *et al*. [19] also reported a heterologous production of Lcc4 from *Aspergillus oryzae* with a comparable activity, but it required longer cultivation and more complicated genetics. In contrast, the current *P. pastoris* expression platform requires shorter cultivation and simpler genetic manipulations and thus it represents a practical system for protein engineering [33].

Lcc5 demonstrated the least thermostability though most of its enzymatic properties (*i.e.,* kinetic parameters and pH stability) were found to be intermediate in comparison with Lcc4A and Lcc7 (Table 2 and Table 3). To date, there is scarce information on the thermostability of laccases, and no single biochemical factor governing it can be concluded [34]. The lowest proline content of Lcc5 (5.3%, whereas others ranged 6.8–8.2%) might be a cue to investigations on its reduced thermostability [35]. Nevertheless, detailed comparative thermostability studies are required to fill the existing knowledge gap on the thermostability of fungal laccases.

The five recombinant laccases possessed five to seven potential *N*-glycosylation sites (five for Lcc4; six for Lcc1A, Lcc1B, and Lcc7; seven for Lcc5) (Fig. 2), falling within the typical count of three to ten [1]. It was generally recognized that glycosylation enhances the stability of fungal laccases by virtue of stabilizing the Cu centers, protecting against proteolysis, and improving thermostability [1]. In the present study, no correlation between the number of potential *N*-glycosylation sites and the thermostability can be observed and two reasons are proposed. First, not all potential sites were glycosylated. The *N*-glycosylation sites were predicted by identifying Asn-X-Ser/Thr motifs against the primary amino acid sequences and did not consider tertiary structure and steric hindrance. Second, the glycosylation patterns between laccases can be different. Glycan moieties of different sugar composition can be conjugated to the Asn-X-Ser/Thr motifs on laccases, resulting different glycan content despite an identical number of *N*-glycosylation sites. However, it is worth mentioning that high carbohydrate content is not necessarily leading to higher laccase thermostability [36]. The exact roles of glycosylation on laccase properties still remain elusive and the current heterologous expression platform should ease exploring the roles through *N*-glycosylation site modification studies [1].

### Comparisons of Bioremediation Performance Induced Re-thinking of the Roles of Laccase in Laccase-mediator Systems (LMSs) and Doubted the Appropriateness of ABTS-based Screenings

**Figure 4:**
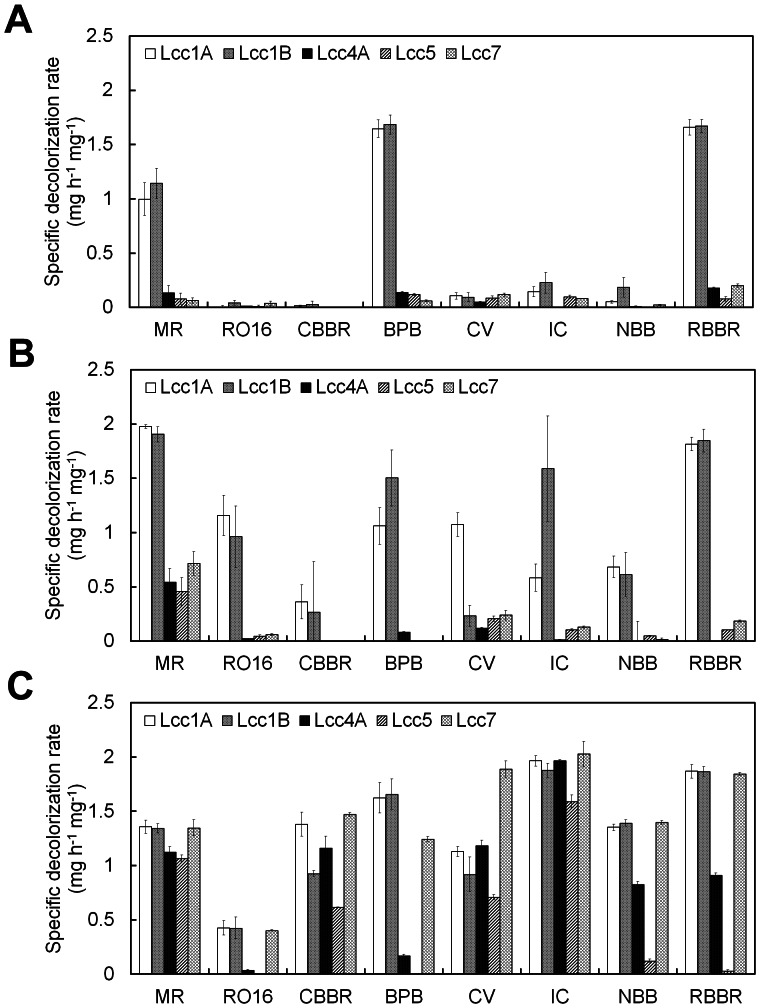
Specific dye decolorization rate of (A) the recombinant laccases, (B) their laccase-HBT system, and (C) laccase-TEMPO system. The reaction was performed in 1×McIlvanie buffer (pH 4) at 30°C by using 5 µg of enzyme with or without 1 mM HBT or TEMPO. The specific decolorization rate was defined as the amount of dye decolorized in one hour by one mg of protein under the assay condition. Detailed decolorization profiles are displayed in Fig. S7. Results shown are the average of three independent experiments ± S.D.

Lcc4A, Lcc5, and Lcc7 formed functional LMSs for dye decolorization and PAH degradation. Eight synthetic dyes from four structural groups and three PAHs with an increasing number of benzene rings were chosen (Fig. S1). Unlike Lcc1A and Lcc1B, the laccase-TEMPO system of Lcc4A, Lcc5, and Lcc7 could achieve higher dye decolorization rates and wider substrate spectra than their corresponding laccase-HBT systems (Fig. 4 and Fig. S7). In fact, mediation by HBT involves a radical ion exchange generating a highly reactive nitroxyl radical (>N–O•) that subsequently oxidizes substrates via hydrogen atom transfer (HAT) mechanism [37]. On the other hand, TEMPO is oxidized to a less reactive oxoammonium ion (>N = O^+^) that subsequently follows a non-radical-ionic mechanism [37]. The results revealed the preference of Lcc4A, Lcc5, and Lcc7 to TEMPO in dye decolorization, differing from the previous Lcc1A and Lcc1B which preferred HBT. Discrepancies between the catalytic mechanisms of these isozymes could be a potential explanation yet further in-depth structure-function comparisons are needed to delineate the scenario.

**Figure 5:**
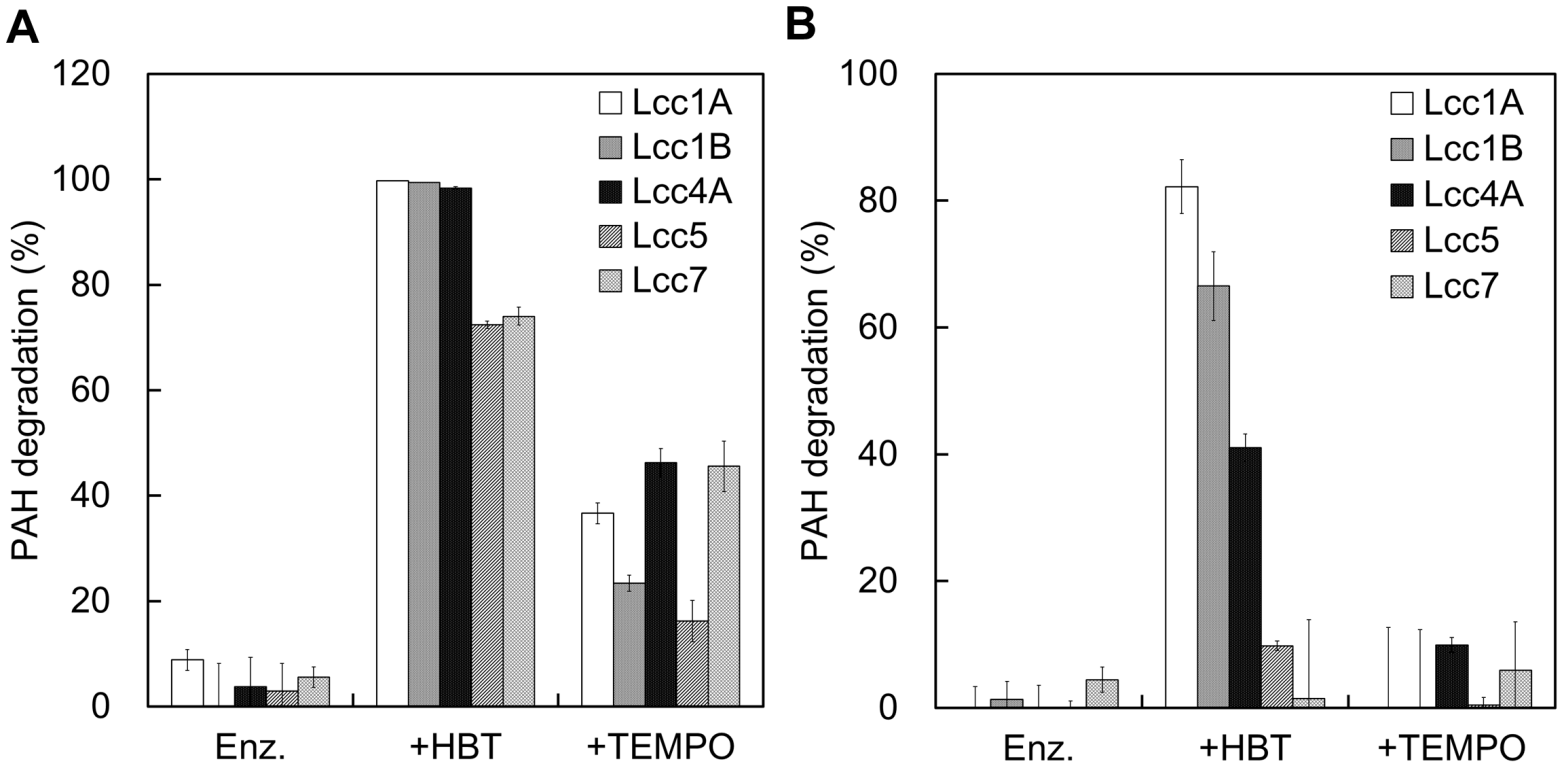
Biodegradation of (A) ANT and (B) BaA by the recombinant laccases and their LMSs. The reaction was performed in 1×McIlvanie buffer (pH 4) at 30°C by using 5 µg of enzyme with or without 1 mM HBT or TEMPO. Residual amount of PAHs was resolved by high-performance liquid chromatography (Waters) equipped with a C-18 reverse-phase column (4.6 mm×250 mm, Grace) at ambient temperature and quantified by a photodiode array detector at 252 nm. Results shown are the average of three independent experiments ± S.D.

This study also revisited the roles of the protein part (*i.e.,* laccases) in LMSs. In general, the oxidized mediators (>N–O• from HBT and >N = O^+^ from TEMPO) in LMSs are believed to react with the recalcitrant substrates following different mechanisms [37]. Laccases are responsible for mediator oxidation but their interaction with the recalcitrant substrates (dyes and PAHs) has not drawn much attention. Indeed, the dye decolorization of Lcc5-TEMPO induced a re-thinking of the role of the protein part and the straight chain mode of oxidizing reactions (laccase oxidizes mediator, then mediator oxidizes dye) in LMSs. The Lcc5-TEMPO system decolorized six of the eight synthetic dyes but left BPB and RO16 colored even after 24 h (data not shown). We proposed two reasons to explain this observation: (i) Lcc5 in the LMS needed to interact with the dyes, in addition to the mediator, to achieve decolorization; (ii) BPB and RO16 specifically inhibited the oxidation of TEMPO by Lcc5. Both reasons suggested that the interaction between laccases and dyes might contribute to the decolorization. The former reason was in favor when further considering the poor dye decolorization by Lcc4A-HBT, Lcc5-HBT, and Lcc7-HBT (Fig. 4 and Fig. S7). The same LMSs demonstrated good ANT degradation (>70%) (Fig. 5), suggesting that the oxidation of HBT was actually not an issue to the three laccases. In addition, the decolorizing ability of the nitroxyl radical from HBT was not questionable as evidenced by the respective LMSs of Lcc1A and Lcc1B and in other studies [11], [38]. Thus, the three laccases might somehow lack well-coordinated interaction with both HBT and dyes, and thus resulted in poor dye decolorization by their lacccase-HBT systems. As a result, it is suggested that a good coordinated interaction between laccase, mediator and dye may be important to efficient dye decolorization by LMSs. In short, the roles of laccase in LMSs could be more than just mediator oxidation.

To our surprise, Lcc7 did not demonstrate the highest decolorization rate compared to the others, irrespective of its highest catalytic efficiency on ABTS (Table 2 and Fig. 4). This suggested that the ABTS activity did not correlate with the decolorization and a more indicative benchmark substrate should be sought. Similarly, Lcc7 also did not perform the best in the biodegradation of PAHs (Fig. 5), and this echoed with the need for a more indicative benchmark substrate to associate with the bioremediation of these toxicants. While ABTS has been recruiting to screen for laccase activity for decades, the appropriateness of using it to indicate diverse applications substrates remains unaddressed. Thus, the results here induced our subsequent investigations to try correlate laccase activity on benchmark substrates, dye decolorization and PAH degradation.

### GUA can be a More Indicative Substrate to Drive Laccase Engineering Towards “Green” Applications

Correlations between the catalytic activity of recombinant laccases on benchmark substrates, dye decolorization and PAH degradation are shown in Table 4. For dye decolorization, the temporal spectroscopic measurements allowed us to calculate the initial dye decolorization rate for correlation analyses. For PAH degradation, since it occurred in a slower manner, we adopted the 24-h incubation described by Alcalde *et al.* [39] and recruited the residual amount of PAH after incubation for analyses. Since chemical factors like pH, ionic strength and radical concentration also influence laccase activity, this study had defined a standard laccase assay condition on both benchmark and application substrates (*i.e.*, 1×McIlvaine buffer, pH 4, 30°C) to allow fair comparisons. Indeed, the correlations had never been addressed despite engineers continuing to employ ABTS and/or DMP as a “direction” for molecular evolution of laccases [6], [7], [8], [10]. The appropriateness of using these screening substrates remained questionable unless clear correlations were shown [13]. The results here clearly revealed that GUA had high association with the decolorization of multiple structurally different dyes (Table 4) and can be a more indicative substrate for the directed evolution of laccases. In fact, dyes, PAHs and other environmental xenobiotics rarely occur individually at a high purity in environment [15], [16], [17]. Thus, a laboratory protein engineering of laccases for bioremediation purposes should seek a surrogate substrate that can indicate diverse xenobiotics. The wider indication spectrum of GUA had made it a more advantageous candidate than ABTS and DMP to direct the protein engineering of laccases toward the realistic bioremediation.

**Table 4:** Correlation^a^ of activity on benchmark substrates, dye decolorization^b^ and PAH degradation^c^.

Laccase or LMS	Application substrate^d^	ABTS	CAT	DMP	DOPA	GUA	SGZ
Laccase	MR	−0.18	−0.32	0.41	−0.28	**0.99**	0.06
	BPB	−0.24	−0.38	0.35	−0.30	**0.96**	0.04
	CV	0.60	−**0.96**	0.53	−**0.90**	−0.06	0.73
	RBBR	−0.19	−0.43	0.38	−0.35	**0.95**	0.09
Laccase-HBT^e^	MR	−0.18	−0.42	0.40	−0.36	**0.93**	0.12
	RO16	−0.27	−0.37	0.31	−0.28	**0.94**	0.02
	CBBR	−0.03	−0.32	0.55	−0.36	**0.97**	0.20
	BPB	−0.18	−0.33	0.41	−0.28	**0.98**	0.06
	CV	−0.07	−0.43	0.28	−0.42	0.49	0.23
	IC	−0.01	−0.31	0.51	−0.31	**0.92**	0.15
	NBB	−0.23	−0.39	0.35	−0.32	**0.94**	0.06
	RBBR	−0.19	−0.39	0.40	−0.33	**0.95**	0.09
	ANT	−0.53	−0.52	−0.18	−0.13	0.58	−0.28
	BaA	−0.53	−0.41	−0.03	−0.13	0.79	−0.23
Laccase-TEMPO^e^	MR	0.40	−0.81	0.73	−0.87	0.55	0.68
	RO16	0.38	−0.62	0.82	−0.74	0.76	0.60
	CBBR	0.57	−0.84	0.85	−**0.93**	0.50	0.79
	BPB	0.33	−0.70	0.77	−0.77	0.74	0.59
	CV	0.80	−0.71	0.51	−0.81	−0.45	0.82
	IC	0.24	−**0.97**	0.33	−0.75	0.20	0.45
	NBB	0.45	−**0.92**	0.71	−**0.91**	0.47	0.69
	RBBR	0.49	−0.88	0.77	−**0.92**	0.51	0.73
	ANT	0.37	−0.77	0.08	−0.61	−0.47	0.45

a Good (|C| ≥0.9) and fair association (0.5<|C|<0.9) were bold and underlined, respectively.

b Initial decolorization rate.

c Degradation % at 24 hours.

d Applications substrates without decolorization/degradation were excluded.

e 1 mM mediator.

**Figure 6:**
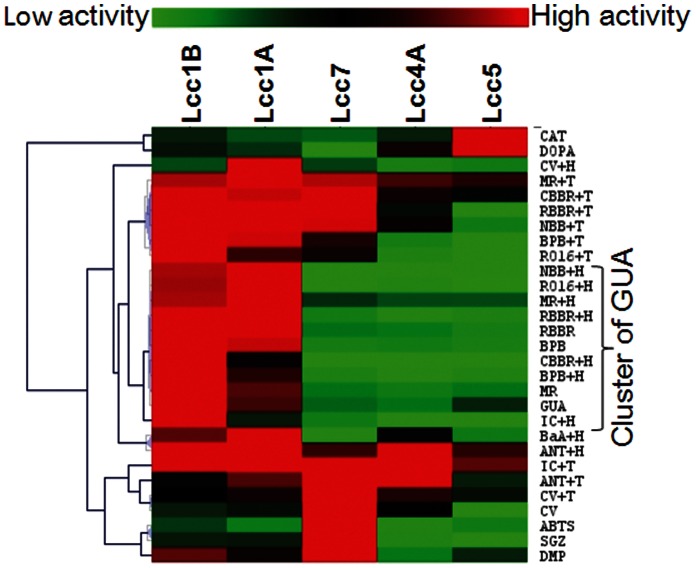
A heat map displaying the association between activity on benchmark substrates, dyes, and PAHs. Clustering was performed by MeV v4.8 based on the Pearson’s correlation, as shown in Table 4. +H: with 1 mM HBT; +T: with 1 mM TEMPO.

The heat map further showed that the activity on GUA well associated with the performance of both laccases and laccase-HBT systems on dye decolorization (Fig. 6). In contrast, activity on ABTS and DMP only fairly associated with dye decolorization although they seemed to partially complement the inadequacy of GUA in indicating the performance of laccase-TEMPO systems (Table 4). The similar chemical mechanism of GUA and HBT oxidation could be a possible reason for the good indication of GUA to laccase-HBT systems (Table 5). In fact, the one-electron oxidation of GUA (a phenolic) involves generation of phenoxyl radical species followed by co-oligomerization [37], [40], [41]. Studies on laccase-catalyzed oxidation of phenol red (another phenolic) had shown that the phenoxyl radical generated by laccases acted analogously to the nitroxyl radical from HBT and both types of radical followed an HAT mechanism [37], [42]. On the contrary, oxidation of ABTS is very different and it takes an electron transfer mechanism to form an ABTS^+•^ radical (Table 5) [37], [43]. Thus, the good indication of GUA here also echoed with the similarity between the oxidation mechanism of phenolics and HBT. However, DMP, another phenolic differs from GUA by an extra *ortho*-methoxy group (Fig. S1), did not show such association with the laccase-HBT systems. Detailed mechanistic studies are needed to unravel the effects of *ortho*-methoxy groups of phenolics on laccase catalysis.

**Table 5:** Chemical mechanism of laccase-catalyzed oxidation of different substrates.

Substrate	Mechanism	Reference
GUA	Generation of phenoxyl radical followed by co-oligomerization	[41]
ABTS^a^	Generation of ABTS^+•^ and then ABTS^2+^; mediation via an electron transfer mechanism	[37], [43]
HBT^a^	Generation of nitroxyl radical (>N–O•); mediation via a hydrogen atom transfer mechanism	[37], [43]
TEMPO^a^	Generation of oxoammonium ion (>N = O^+^); mediation via a non-radical-ionic mechanism	[37], [43]
Azo dyes	Abstract of one electron to generate phenoxyl radical; abstract of a second electron to yield an aromatic cation	[48]
Triarylmethane dyes	Successive demethylation/deethylation by laccases; hydroxylation of dye to form carbinol followed by successive oxidation by LMSs	[49]
Indigoid dyes	Formation of dehydroindigo followed by nucleophilic attack by water molecules	[50]
Anthraquinone dyes	Single electron oxidation of secondary amine to imine followed by nucleophilic attack by water molecules	[51], [52]

a Laccase mediators.

Comparisons of the oxidation mechanism of GUA and azo dyes showed that they both involve generation of phenoxyl radicals (Table 5). Although this can be a cue to further investigate the correlation of their oxidation mechanisms, it should not be the only direction as other dyes which well associated with GUA activity actually did not recruit phenoxyl radicals in decolorization (Table 5). The redox potential of laccase is another direction that warrants further investigations because it is known to correlate with laccase activity on ABTS [1], [4], [7]. However, its correlation with the wide spectrum of laccase substrates remains opaque and a comprehensive analysis on it will provide useful insights into the electrochemistry of laccases.

In fact, GUA had been overlooked and, to the best of our knowledge, it has not yet been employed as a screening substrate in any engineering study of laccases. Considering the technical perspectives, well-proven applications of laccase-HBT systems (*e.g*., Lignozym® process) on lignin degradation and on bioremediations further highlighted the industrial relevance of employing GUA as a screening substrate [5], [38], [44]. Another intrinsic advantage of GUA was its natural origin from lignin so that its high catalytic activity could be further associated with efficient degradation of lignocellulose. In fact, ABTS and DMP clustered closely in the heat map, and this suggests that they associated well with each other (Fig. 6). As a result, dual substrate screening which used ABTS and DMP seemed redundant to the molecular evolution of laccases for bioremediation. In addition, the standard assay condition employed here allowed fair comparisons of laccase activities on different substrates. However, chemical factors (*e.g*., pH, ionic strength, and radical concentration) may affect the correlation of substrates. Thus, adapting the standard GUA assay in this study can secure the correlation and direct laccase engineering to bioremediation applications. Finally, oxidized GUA has a molar extinction coefficient of 12,000 M^−1^ cm^−1^ at optimal wavelength (470 nm) which is ∼threefold smaller than that of ABTS and DMP [2]. However, this does not detrimentally affect the screening sensitivity of GUA. This is because increasing the light path would provide an easy compensation during high-throughput screenings in the course of directed evolution.

### Conclusions

This study demonstrated the high robustness of *P. pastoris* for heterologous expression of a number of “difficult-to-express” laccases. The platform enabled individual expression and characterizations of five *L. edodes* laccases. Lcc7 represented a novel laccase demonstrating the highest catalytic efficiency (on ABTS) and thermostability, and it could serve as a starting candidate for engineering. Comparative enzymology, correlation and hierarchical clustered analyses suggest GUA to be an indicative benchmark substrate to direct the molecular evolution of laccases towards decolorization of multiple dyes, whereas the commonly employed ABTS and DMP did not achieve such an indication. In conclusion, this study provided a robust heterologous yeast expression platform and an indicative screening substrates, GUA, which are the major prerequisites to engineer laccase towards more efficient “green” applications.

## Supporting Information

Figure S1 **Chemical structure of benchmark substrates, synthetic dyes, PAHs, and mediators employed in this study.**
(TIF)

Figure S2 **Amino acid sequence alignments of allelic forms of Lcc4.** Substitutions are in black on a white background.
(TIF)

Figure S3 **Effects of temperature on Lcc4A (•), Lcc5 (▴), and Lcc7 (▪).** Results of Lcc1A (⧫) and Lcc1B (◊) are included for comparison. (A) Thermostability of the recombinant enzymes incubated at desired temperature for 30 minutes. (B) Time-dependent thermostability at 50°C. Residual activity of enzymes without heat treatment was taken as 100%. Assays were performed in 1×McIlvanie buffer (pH 4) by using 1 mM ABTS at 30°C. Results shown are the average of three independent experiments ± S.D.
(TIF)

Figure S4 **Effects of co-solvents on the activity of Lcc4A (•), Lcc5 (▴), and Lcc7 (▪).** Results of Lcc1A (⧫) and Lcc1B (◊) are included for comparison. (A) ACE: acetone; (B) ACN: acetonitrile; (C) EtOH: ethanol; (D) MeOH: methanol. Residual activity of enzymes in solvent-free condition was taken as 100%. Assays were performed in 1×McIlvanie buffer (pH 4) by using 1 mM ABTS at 30°C in the presence of respective solvent. Results shown are the average of three independent experiments ± S.D.
(TIF)

Figure S5 **Homology model of Lcc7 (blue) superimposed with that of Lcc1A (pink).** The four coordinated Cu atoms are in brown. An extra loop (V392-P399) at the entrance of the active site of Lcc7 is red-boxed.
(TIF)

Figure S6 **Effects of pH on Lcc4A (•), Lcc5 (▴), and Lcc7 (▪).** Results of Lcc1A (⧫) and Lcc1B (◊) are included for comparison. (A) Stability of the recombinant enzymes incubated at desired pH for 30 minutes before assaying with 1 mM ABTS in 1×McIlvanie buffer (pH 4) at 30°C. (B) Dependence of activity at different pH. Assays were performed in 1×McIlvanie buffer (pH 2–8) by using 1 mM ABTS at 30°C. Relative activity was defined as 100% at respective optimal pH. Results shown are the average of three independent experiments ± S.D.
(TIF)

Figure S7 **Dye decolorization by (A) Lcc4A (•), Lcc5 (▴), Lcc7 (▪), Lcc1A (⧫), and Lcc1B (◊), (B) their laccase-HBT system, and (C) laccase-TEMPO system.** The reaction was performed in 1×McIlvanie buffer (pH 4) at 30°C by using 5 µg of enzyme with or without 1 mM HBT or TEMPO. Residual amount of dyes was followed spectrophotometrically at the optimal wavelength. Results shown are the average of three independent experiments ± S.D.
(TIF)

Table S1 **Sequence of specific primers for cloning individual laccase isozymes of** ***L. edodes*** **L54.**
(DOC)
