# Supplementary figures and images for: A Novel Lentinula edodes Laccase and Its Comparative Enzymology Suggest Guaiacol-Based Laccase Engineering for Bioremediation
Source: PLoS One. 2013 Jun 14;8(6):e66426. doi: 10.1371/journal.pone.0066426 (PMC3683064; doi:10.1371/journal.pone.0066426)

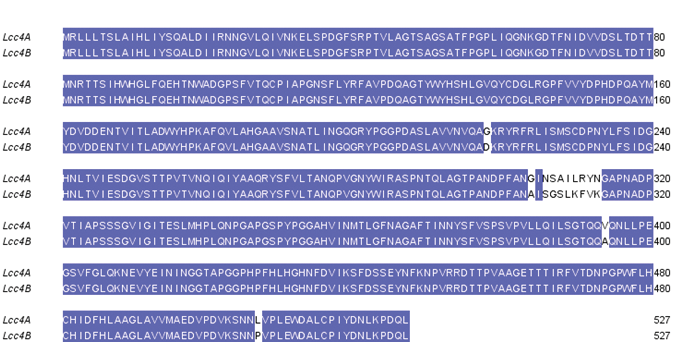

Supplement: Figure S2 — Amino acid sequence alignments of allelic forms of Lcc4. Substitutions are in black on a white background. (TIF) [file pone.0066426.s002.tif]

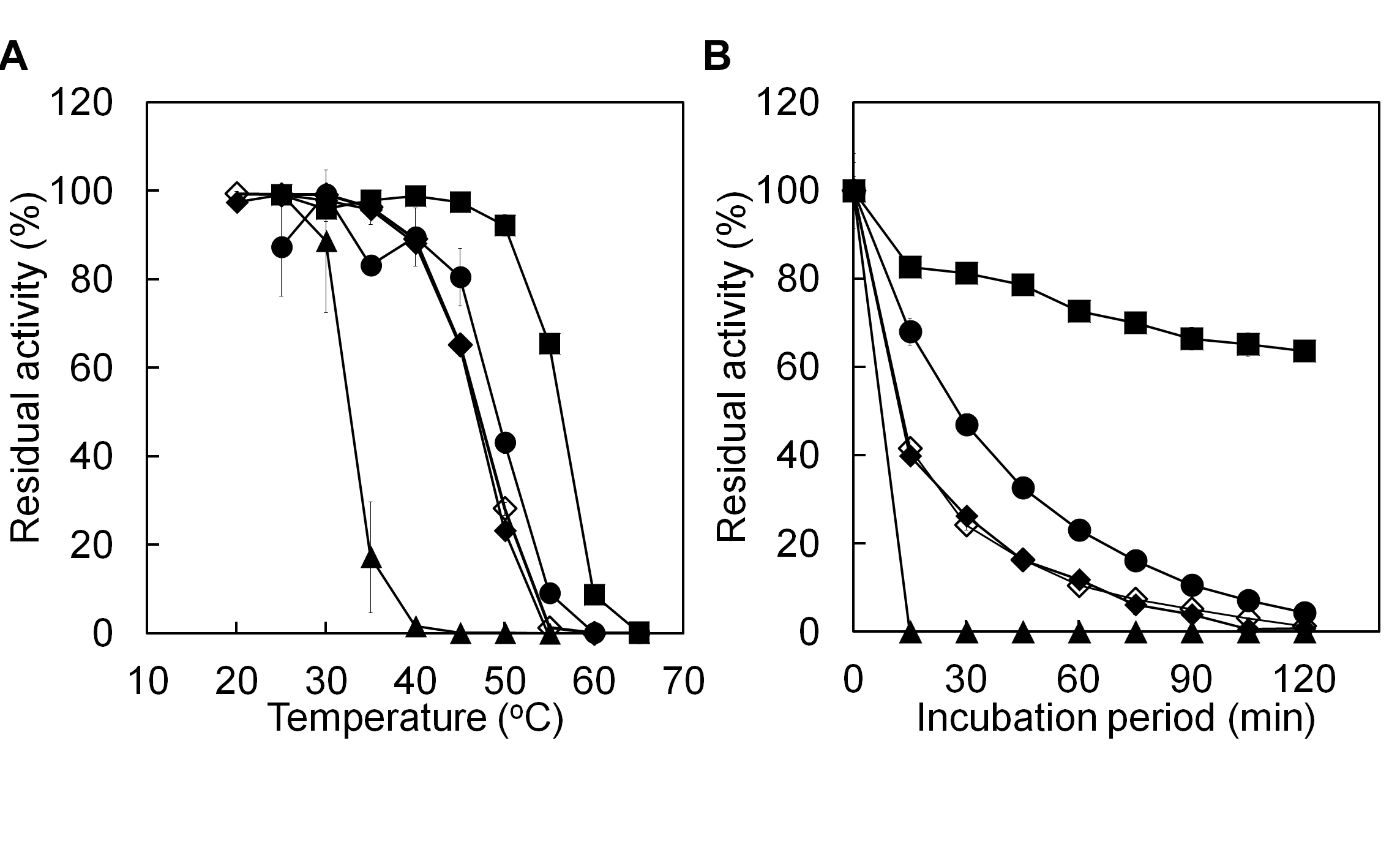

Supplement: Figure S3 — Effects of temperature on Lcc4A (•), Lcc5 (▴), and Lcc7 (▪). Results of Lcc1A (⧫) and Lcc1B (◊) are included for comparison. (A) Thermostability of the recombinant enzymes incubated at desired temperature for 30 minutes. (B) Time-dependent thermostability at 50°C. Residual activity of enzymes without heat treatment was taken as 100%. Assays were performed in 1×McIlvanie buffer (pH 4) by using 1 mM ABTS at 30°C. Results shown are the average of three independent experiments ± S.D. (TIF) [file pone.0066426.s003.tif]

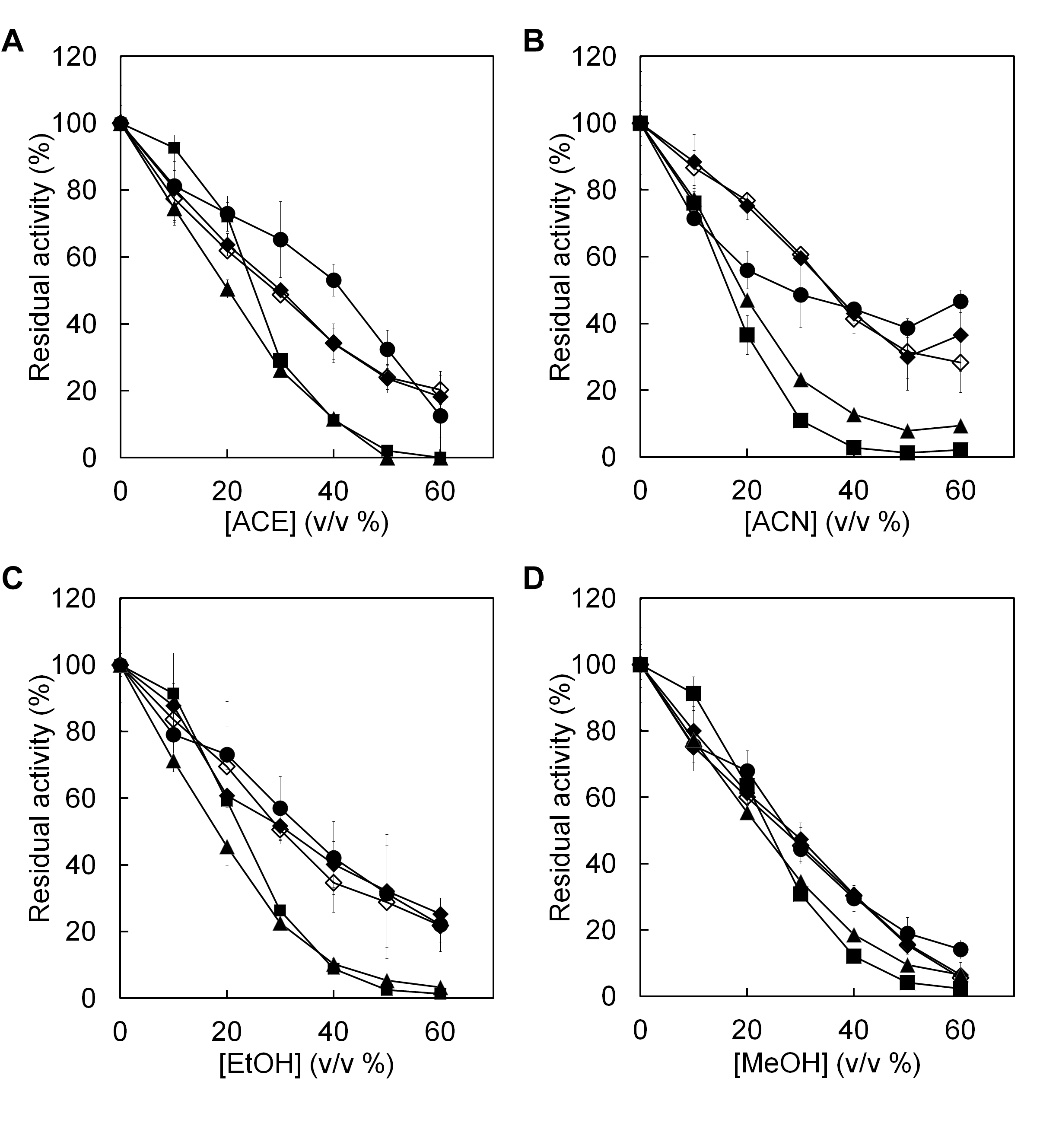

Supplement: Figure S4 — Effects of co-solvents on the activity of Lcc4A (•), Lcc5 (▴), and Lcc7 (▪). Results of Lcc1A (⧫) and Lcc1B (◊) are included for comparison. (A) ACE: acetone; (B) ACN: acetonitrile; (C) EtOH: ethanol; (D) MeOH: methanol. Residual activity of enzymes in solvent-free condition was taken as 100%. Assays were performed in 1×McIlvanie buffer (pH 4) by using 1 mM ABTS at 30°C in the presence of respective solvent. Results shown are the average of three independent experiments ± S.D. (TIF) [file pone.0066426.s004.tif]

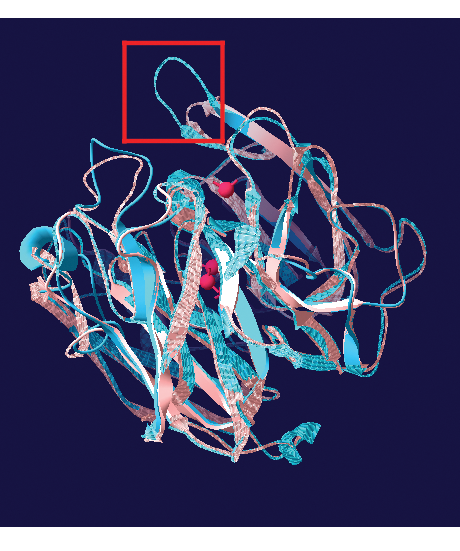

Supplement: Figure S5 — Homology model of Lcc7 (blue) superimposed with that of Lcc1A (pink). The four coordinated Cu atoms are in brown. An extra loop (V392-P399) at the entrance of the active site of Lcc7 is red-boxed. (TIF) [file pone.0066426.s005.tif]

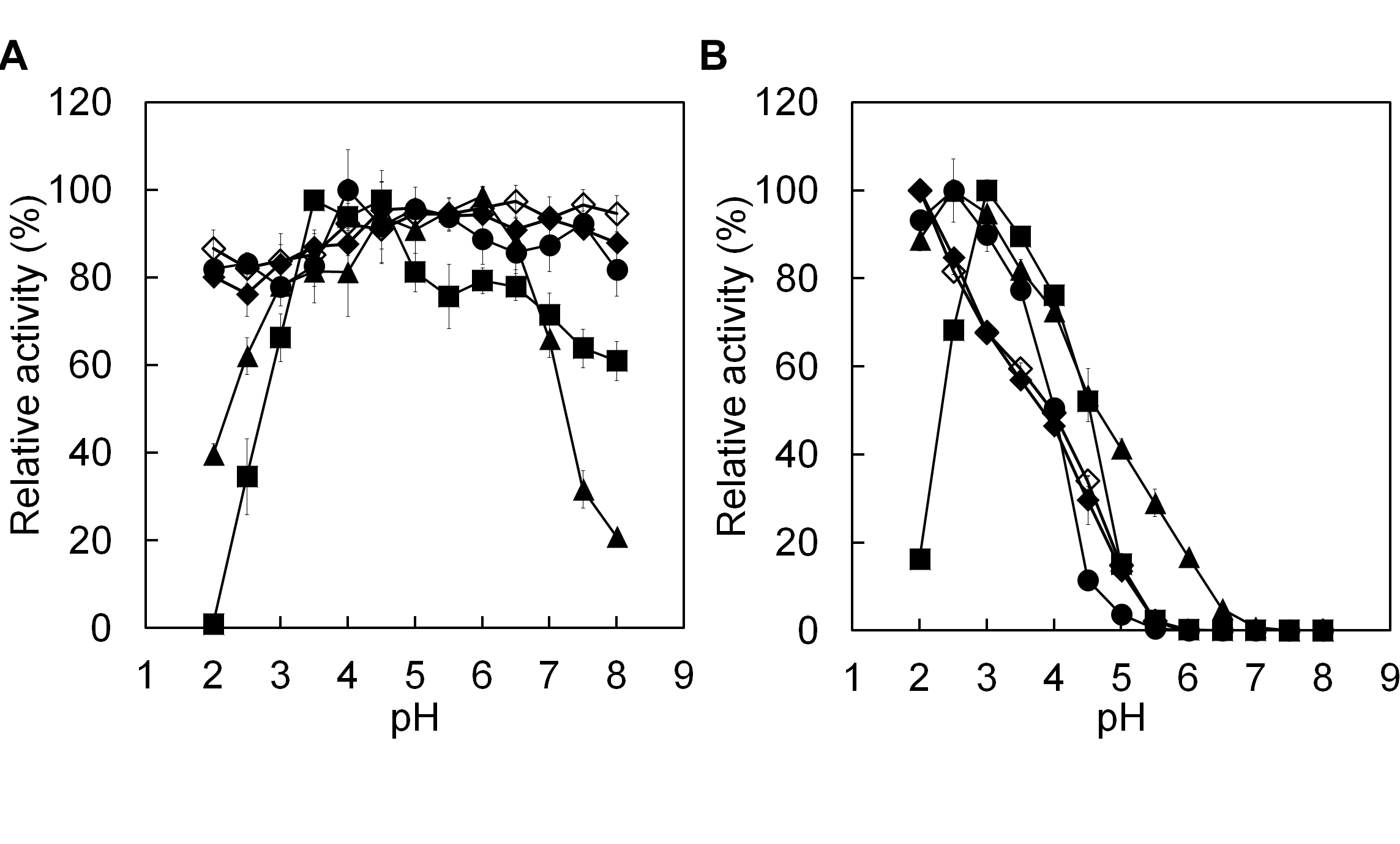

Supplement: Figure S6 — Effects of pH on Lcc4A (•), Lcc5 (▴), and Lcc7 (▪). Results of Lcc1A (⧫) and Lcc1B (◊) are included for comparison. (A) Stability of the recombinant enzymes incubated at desired pH for 30 minutes before assaying with 1 mM ABTS in 1×McIlvanie buffer (pH 4) at 30°C. (B) Dependence of activity at different pH. Assays were performed in 1×McIlvanie buffer (pH 2–8) by using 1 mM ABTS at 30°C. Relative activity was defined as 100% at respective optimal pH. Results shown are the average of three independent experiments ± S.D. (TIF) [file pone.0066426.s006.tif]

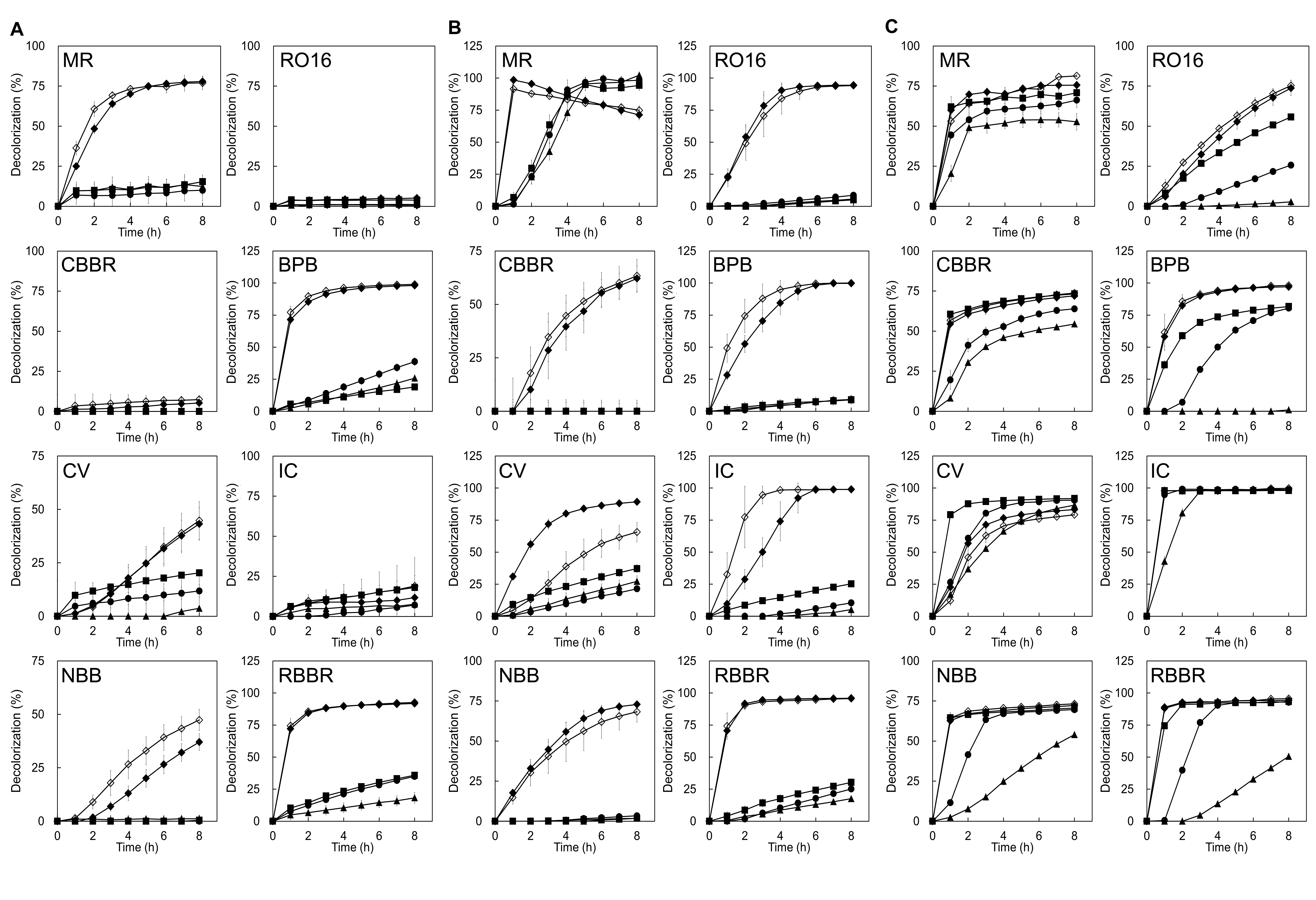

Supplement: Figure S7 — Dye decolorization by (A) Lcc4A (•), Lcc5 (▴), Lcc7 (▪), Lcc1A (⧫), and Lcc1B (◊), (B) their laccase-HBT system, and (C) laccase-TEMPO system. The reaction was performed in 1×McIlvanie buffer (pH 4) at 30°C by using 5 µg of enzyme with or without 1 mM HBT or TEMPO. Residual amount of dyes was followed spectrophotometrically at the optimal wavelength. Results shown are the average of three independent experiments ± S.D. (TIF) [file pone.0066426.s007.tif]
