# Supplementary material for: A Novel Lentinula edodes Laccase and Its Comparative Enzymology Suggest Guaiacol-Based Laccase Engineering for Bioremediation
Source: PLoS One. 2013 Jun 14;8(6):e66426. doi: 10.1371/journal.pone.0066426 (PMC3683064; doi:10.1371/journal.pone.0066426)
Supplement: Table S1 — Sequence of specific primers for cloning individual laccase isozymes of L. edodes L54. (DOC) [file pone.0066426.s008.doc]

**Table S1.** Sequence of specific primers for cloning individual laccase isozymes of *L. edodes* L54

| Primer | Sequence (5’→3’)^a^ |
| --- | --- |
| Lcc2-F | ATGCTTCCCTTCGTTTATCTTCTGG |
| Lcc2-R | TCAAGGTAATTGAGCAGGGGTTAAG |
| Lcc3-F | ATGTTTCTTATCAACGTTGTCTTCGG |
| Lcc3-R | CTAAGGTAGTTGTGAAGGGGTCAAAC |
| Lcc4-F | ATGCGTTTACTCTTGACTTCTCTCGC |
| Lcc4-R | TCAAAGCTGGTCGGGCTTGA |
| Lcc5-F | ATGGGCTTTCAATCTTTTACCTCCC |
| Lcc5-R | TCAAAGTTGGTCAGCAGAAAGAGC |
| Lcc7-F | ATGTTCAAGATCAAATTTGCTCTCGA |
| Lcc7-R | TCAAGGCAGTTGATCAGGAGTCAA |
| Lcc8-F | ATGAAGTTCCTAGTTGCAACCGCT |
| Lcc8-R | TCATTGCAAGTTCGCAGGAAGAG |
| Lcc9-F | ATGAAACTCCTAGCGTCTACCGTTCT |
| Lcc9-R | TCACTGCAAATCAGGTGAAAGAGC |
| Lcc10-F | ATGCAGCCGGCGTCTGTCT |
| Lcc10-R | TCATGCAGTTGGCTTAATCAGTCC |
| Lcc11-F | ATGGGCACAAGGCTTTCTACCTG |
| Lcc11-R | TTAGGAACAACTATTTGCCGCATTTAT |
| Lcc2-F-*Avr*II | TTACCTAGGACCATGCTTCCCTTCGTTTATCTTCTGGCGG |
| Lcc2-R-*Not*I | TAAGCGGCCGCTCAAGGTAATTGAGCAGGGGTTAAGCTGT |
| Lcc3-F-*Avr*II | TAACCTAGGACCATGTTTCTTATCAACGTTGTCTTCGGATC |
| Lcc3-R-*Not*I | TAAGCGGCCGCCTAAGGTAGTTGTGAAGGGGTCAAACTATTATATAT |
| Lcc4-F-*Avr*II | TTACCTAGGACCATGCGTTTACTCTTGACTTCTCTCGCTATC |
| Lcc4-R-*Not*I | TAAGCGGCCGCTCAAAGCTGGTCGGGCTTGAGATTATCATAA |
| Lcc5-F-*Avr*II | TTACCTAGGACCATGGGCTTTCAATCTTTTACCTCCCTCC |
| Lcc5-R-*Not*I | TAAGCGGCCGCTCAAAGTTGGTCAGCAGAAAGAGCGTCGT |
| Lcc7-F-*Avr*II | TTACCTAGGACCATGTTCAAGATCAAATTTGCTCTCGAACC |
| Lcc7-R-*Not*I | TAAGCGGCCGCTCAAGGCAGTTGATCAGGAGTCAAACTATC |
| Lcc8-F-*Avr*II | TTACCTAGGACCATGAAGTTCCTAGTTGCAACCGCTTCTG |
| Lcc8-R-*Not*I | TAAGCGGCCGCTCATTGCAAGTTCGCAGGAAGAGCAT |
| Lcc9-F-*Avr*II | TTACCTAGGACCATGAAACTCCTAGCGTCTACCGTTCTGG |
| Lcc9-R-*Not*I | TAAGCGGCCGCTCACTGCAAATCAGGTGAAAGAGCATCAT |
| Lcc10-F-*Avr*II | TTACCTAGGACCATGCAGCCGGCGTCTGTCTTTCTTCTGC |
| Lcc10-R-*Not*I | TAAGCGGCCGCTCATGCAGTTGGCTTAATCAGTCCGAAC |
| Lcc11-F-*Avr*II | TTACCTAGGACCATGGGCACAAGGCTTTCTACCTGGAAAT |
| Lcc11-R-*Not*I | TAAGCGGCCGCTTAGGAACAACTATTTGCCGCATTTATCCT |

^a^ Restriction sites were underlined
